# Optimal diagnostic method using multidetector-row computed tomography for predicting lymph node metastasis in colorectal cancer

**DOI:** 10.1186/s12957-019-1583-y

**Published:** 2019-02-22

**Authors:** Tsutomu Kumamoto, Junichi Shindoh, Hideaki Mita, Yuriko Fujii, Yuichiro Mihara, Michiro Takahashi, Nobuyuki Takemura, Takako Shirakawa, Hisashi Shinohara, Hiroya Kuroyanagi

**Affiliations:** 1 Department of Gastrointestinal Surgery, JR Tokyo General Hospital, Yoyogi 2-1-3, Shibuya-ku, Tokyo, Japan; 2 Department of Gastroenterological Surgery, Toranomon Hospital, Tranomon 2-2-2, Minato-ku, Tokyo, Japan; 3 Department of Radiology, JR Tokyo General Hospital, Yoyogi 2-1-3, Shibuya-ku, Tokyo, Japan; 4 Present Address: Department of Surgery, Hyogo College of Medicine, Mukogawa-cho 1-1, Nishinomiya, Hyogo 663-8501 Japan

**Keywords:** Colorectal cancer, Lymph node metastasis, Multidetector-row computed tomography, Preoperative diagnosis

## Abstract

**Background:**

Prediction of nodal involvement in colorectal cancer is an important aspect of preoperative workup to determine the necessity of preoperative treatment and the adequate extent of lymphadenectomy during surgery. This study aimed to investigate newer multidetector-row computed tomography (MDCT) findings for better predicting lymph node (LN) metastasis in colorectal cancer.

**Methods:**

Seventy patients were enrolled in this study; all underwent MDCT prior to surgery and upfront curative resection for colorectal cancer. LNs with a short-axis diameter (SAD) ≥ 4 mm were identified on MDCT images, and the following measures were recorded by two radiologists independently: two-dimensional (2D) SAD, 2D long-axis diameter (LAD), 2D ratio of SAD to LAD, 2D CT attenuation value, three-dimensional (3D) SAD, 3D LAD, 3D SAD to LAD ratio, 3D CT attenuation value, LN volume, and presence of extranodal neoplastic spread (ENS), as defined by indistinct nodal margin, irregular capsular enhancement, or infiltration into adjacent structures.

**Results:**

Forty-six patients presented 173 LNs with a SAD ≥ 4 mm, while 24 patients exhibited pathologically confirmed LN metastases. Receiver operating characteristic analysis revealed that 2D LAD was the most sensitive measure for LN metastases with an area under the curve of 0.752 (cut-off value, 7.05 mm). When combined with CT findings indicating ENS, 2D LAD (> or ≤ 7 mm) showed enhanced predictive power for LN metastases (area under the curve, 0.846; *p* < 0.001).

**Conclusions:**

LAD in axial MDCT imaging is the most sensitive measure for predicting colorectal LN metastases, especially when MDCT findings of ENS are observed.

## Background

Colorectal cancer is the third leading cause of cancer-related death worldwide. The presence of lymph node (LN) metastasis is one of the most important factors associated with poor prognosis [1, 2], and preoperative treatment is considered in selected patients who are expected to exhibit prolonged survival [3, 4]. Since preoperative staging is critical in determining treatment sequence and surgical indication, preoperative prediction of nodal involvement is an important aspect of colorectal cancer management.

The LNs measuring > 10 mm in computed tomography (CT) images have conventionally been regarded as a sign of nodal involvement of cancer [5, 6]. Previous studies have reported the diagnostic accuracy of such CT measures, using axial images [5–9]. However, these reports were based on data collected prior to the availability of current high-resolution CT imaging equipment; therefore, the predictive value of CT findings for LN metastasis has not yet been fully validated.

Recently, use of multidetector-row computed tomography (MDCT) has become a routine practice for preoperative diagnosis, as its thinner slice data enable various additional analyses, including three-dimensional (3D) reconstruction. However, it remains unclear how much this novel imaging modality has changed the accuracy of cancer diagnosis.

In this study, we evaluated the diagnostic accuracy of various radiologic measures obtained by newer MDCT data and sought to re-evaluate a sensitive approach to predict nodal involvement in colorectal cancer.

## Methods

### Patients

This study was approved by the Ethics Committee of our hospital. Our prospectively collected colorectal cancer database was analyzed retrospectively. The initial population considered for this study included 95 consecutive patients who underwent preoperative MDCT and resection of colorectal cancer at our institution between April 2016 and February 2018. Of these, the following patients were excluded from the current analysis in order to analyze the pure correlation between CT images and pathologic findings: patients with non-curative resection (*n* = 11), patients who underwent preoperative chemotherapy and/or radiation therapy (*n* = 6), patients for whom the current CT protocol was not followed (*n* = 6), and patients who did not exhibit a one-to-one correlation between detected LNs and dissected LNs (*n* = 2). The remaining 70 patients were studied in detail; all remaining patients had been diagnosed with adenocarcinoma of colorectal origin and had undergone complete mesocolic excision or total mesorectal excision with central vascular ligation [10, 11].

### CT protocol

A 64-detector row CT scanner (OPTIMA CT660PRO; GE Healthcare, Chicago, IL, USA) was used for CT analysis. Protocol CT parameters were as follows: section thickness, 0.625 mm; pitch, 0.984; construction interval, 0.625 mm; noise index, 9.8 (at 5-mm thickness); 120 kV; and tube rotation time, 0.7 s. A total of 60 mg I per kg of non-ionic contrast material was administered intravenously over a period of 30 s, using a power injector (Stellant CT Injection System Stellant D Dural Flow; Bayer Medical Care, Pittsburgh, PA, USA). Imaging was performed at 25 and 85 s after the initiation of contrast material injection, corresponding to the arterial and late phases, respectively.

### Evaluation

**Fig. 1:**
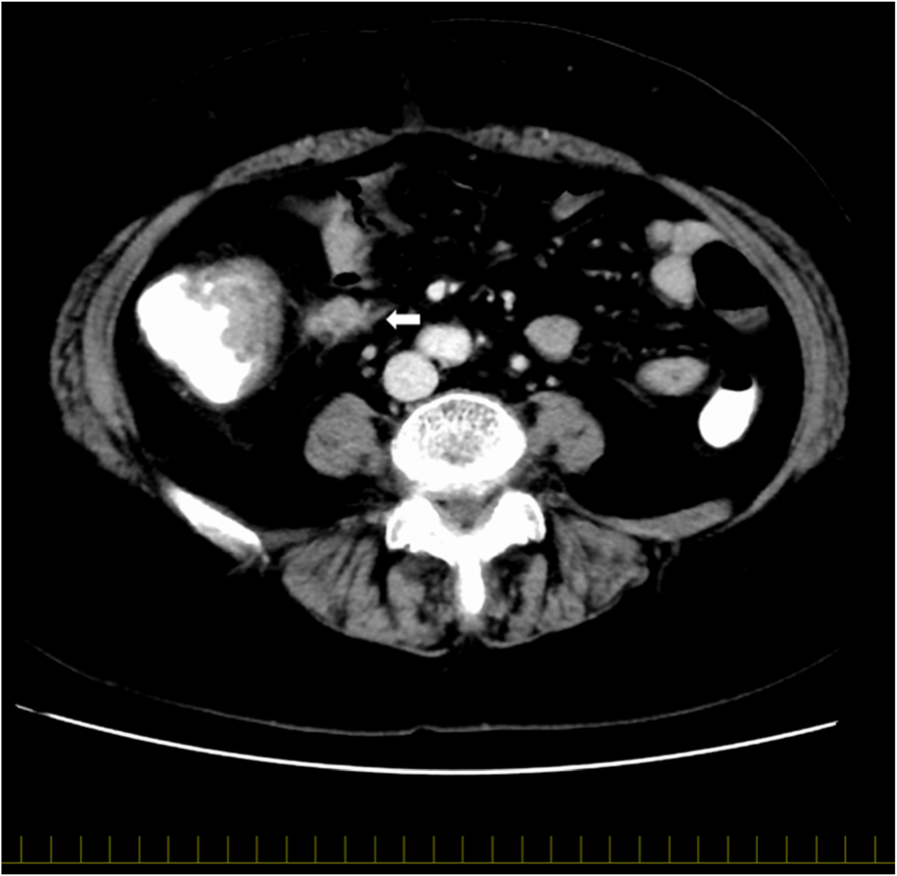
Extranodal neoplastic spread (ENS): the white arrow indicates a lymph node with ENS

**Fig. 2:**
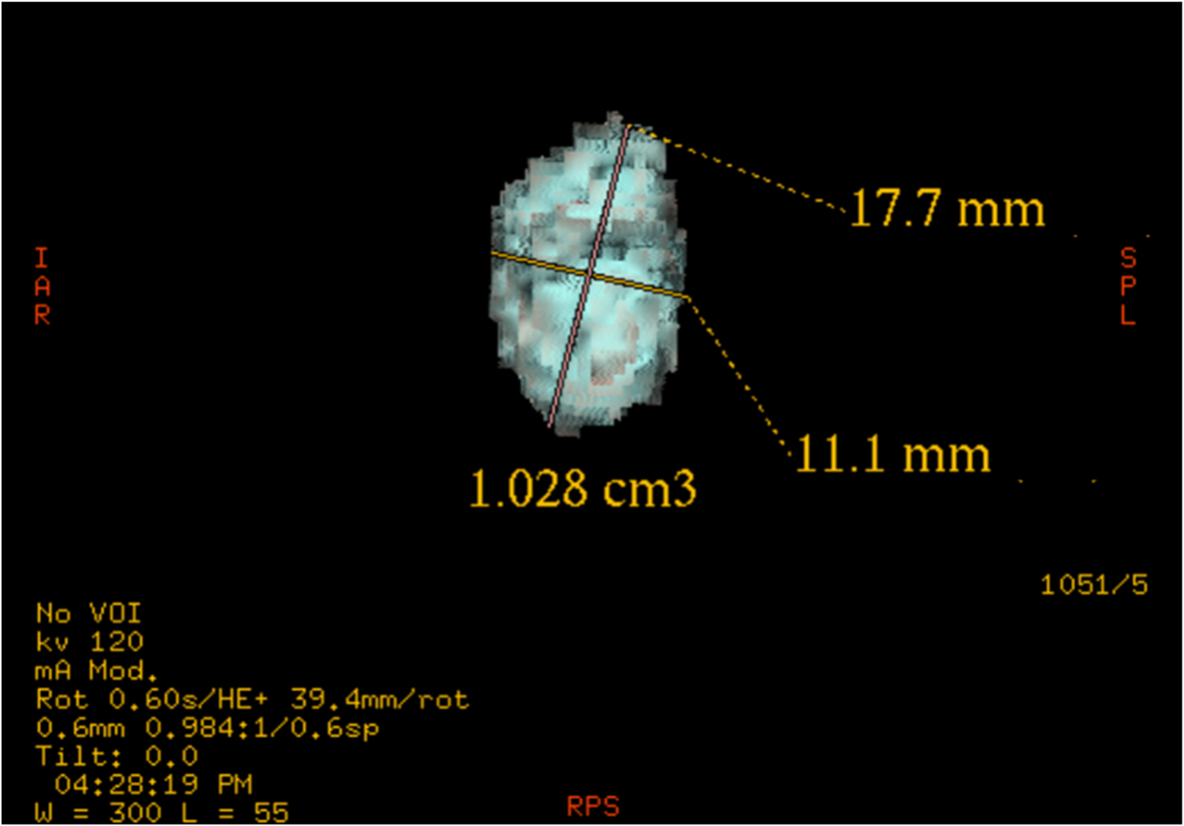
Three-dimensional reconstruction: the size and volume of the lymph node were measured

The LNs were screened on axial MDCT images (0.625-mm-thick slices); the mediastinal window settings consisted of a window level of 55 and a window width of 300, in the late phase. Two experienced radiologists identified enlarged LNs measuring ≥ 4 mm in short-axis diameter (SAD), preoperatively. The following CT measures were recorded in every patient: long-axis diameter (LAD), the ratio of SAD to LAD, and maximum and average CT attenuation values. The presence of indistinct nodal margins, irregular nodal capsular enhancement, or infiltration into the adjacent structure (Fig. 1) were also recorded as signs of “extranodal neoplastic spread” (ENS) [12]. In addition, all LNs were reconstructed in 3D using a workstation (Advantage Workstation Volume Share 4, version 4.5, GE Healthcare, Milwaukee, WI, USA); 3D SAD, 3D LAD, maximum and average CT attenuation values in 3D images, and LN volume were also measured (Fig. 2). For each LN, a one-to-one correlation between the detected and dissected LN was confirmed; the LN was then assessed pathologically.

### Statistical analysis

All statistical analyses were performed using SPSS version 24 (IBM Corp., Armonk, NY, USA). Factors associated with LN enlargement on MDCT were investigated using multivariate logistic regression with backward elimination. The predictive value of LN metastases was measured via area under the curve (AUC) in a receiver operating characteristic curve analysis. The optimal cut-off point in the receiver operating characteristic analysis was determined by Youden’s index (*J*), calculated using the equation *J* = sensitivity + (1 − specificity).

## Results

### Patients and LN status

The baseline characteristics of the patient population are summarized in Table 1. In total, 1644 LNs were dissected from 70 patients (median, 21.5 LNs; range, 4–50 LNs per patient). A total of 173 enlarged LNs were detected in 46 patients. Of these, 56 LNs (32.4%) from 24 (52.2%) patients were histopathologically positive for LN metastasis. In another four patients who did not show LN enlargement on preoperative MDCT, LN metastasis was confirmed pathologically.

**Table 1:** Baseline patient characteristics

Characteristic	Value (*n* = 70)
Age (years)	70 (39–95)
Male/female ratio	41/29
Body mass index (kg/m^2^) (range)	22.6 (14.1–30.5%)
Tumor location	
Appendix vermiformis/cecum	15 (21.4%)
Ascending colon	9 (12.9%)
Transverse colon	8 (11.4%)
Descending colon	3 (4.3%)
Sigmoid colon/rectosigmoid colon	25 (35.7%)
Rectum	10 (14.3%)
Depth of tumor	
Mucosa	4 (5.7%)
Submucosa	14 (20.0%)
Muscularis propria	9 (12.9%)
Subserosa	28 (40.0%)
Exposure on submucosa or invade other organs or structures	15 (21.4%)
Differentiation^a^	
Well differentiated	32 (45.7%)
Moderately differentiated	35 (50.0%)
Poorly differentiated	3 (4.3%)
Obstruction^b^	17 (24.3%)
Preoperative intervention	7 (10.0%)
Endoscopic resection	4 (5.7%)
Ileus tube	3 (4.3%)
WBC (/mm^3^)	5700 (2600–14,700)
NLR	2.40 (0.01–9.65)
CRP (mg/mL)	0.16 (0.01–6.20)
CEA (ng/mL)	3.6 (0.7–98)
CA19-9 (U/L)	6.0 (0.07–4412)
Days from CT to surgery	20 (2–71)
Lymph node enlargement on CT	46 (65.7%)
Pathologically confirmed metastasis	28 (40.0%)

Data are presented as median (range) or number (%) unless otherwise indicated

*WBC* white blood cell, *NLR* neutrophil to lymphocyte ratio, *CRP* c-reactive protein, *CEA* carcinoembryonic antigen, *CA19-9* carbohydrate antigen 19-9, *CT* computed tomography

^a^Dominant histopathological features of differentiation

^b^Obstruction, which was not passed through with an endoscope

### Factors influencing LN enlargement on MDCT

Table 2 shows the results of univariate analysis for factors predicting LN enlargement. Bowel obstruction, increased white blood cell count, and pathologically confirmed metastasis correlated with LN enlargement, as detected on MDCT. Multivariate analysis revealed that pathologically confirmed metastasis was the only predictive factor for LN enlargement (odds ratio, 6.00; 95% confidence interval, 2.08–17.3; *p* = 0.006).

**Table 2:** Univariate analyses of clinical factors predicting lymph node enlargement on multidetector computed tomography

Clinical factor	*p* value
Age	0.556
Male/female ratio	0.630
Body mass index	0.586
Tumor location (right side vs left side)	0.623
Differentiation	0.323
Obstruction	0.025
Ileus tube	0.201
Endoscopic resection	0.077
WBC	0.004
NLR	0.138
CRP	0.204
Pathologically confirmed metastasis	0.004

*WBC* white blood cell, *NLR* neutrophil to lymphocyte ratio, *CRP* c-reactive protein

### Evaluation of LN enlargement on MDCT

**Fig. 3:**
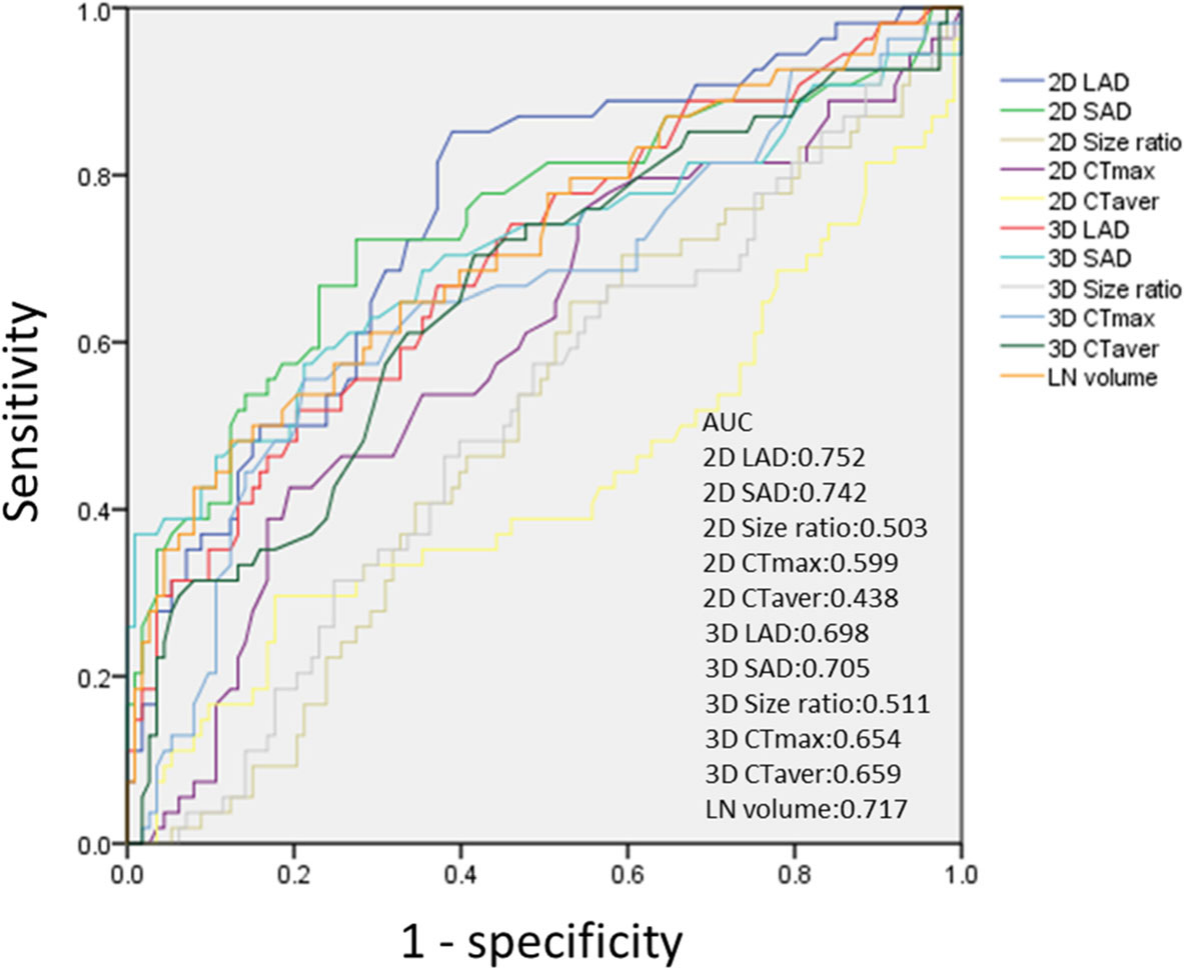
Receiver operating characteristic curves show the ability of each multidetector computed tomography measurement to predict lymph node metastasis. AUC, area under the curve; 2D, two-dimensional; 3D, three-dimensional; SAD, short-axis diameter; LAD, long-axis diameter; CTmax, maximum computed tomography attenuation value; CTaver, average computed tomography attenuation value; LN, lymph node

The comparison of the predictive power of various CT measures for histopathologic nodal involvement of cancer is shown in Fig. 3. The 2D LAD on the axial image showed the highest AUC (0.752), followed by 2D SAD (AUC, 0.742), nodal volume (AUC, 0.717), 3D SAD (AUC, 0.705), 3D LAD (AUC, 0.698), 3D average CT attenuation value (AUC, 0.659), 3D maximum CT value (AUC, 0.654), 2D maximum CT attenuation value (AUC, 0.599), 3D size ratio (AUC, 0.511), 2D size ratio (AUC, 0.503), and 2D average CT attenuation value (AUC, 0.438). The optimal cut-off value of 2D LAD for predicting LN metastases was 7.05 mm, and the sensitivity, 0.821; specificity, 0.624; accuracy, 0.688; positive predictive value (PPV), 0.511; and negative predictive value (NPV), 0.880 were estimated.

Among the 173 enlarged LNs, ENS was observed in 41 (23.7%). The predictive power for LN metastases, focusing on these LNs presenting ENS, was superior to that for the simple morphological enlargement of LNs (sensitivity, 0.643; specificity, 0.957; accuracy, 0.855; PPV, 0.878; and NPV, 0.848).

Based on these results, a new scoring system was developed: 2D LAD (> 7 mm [1 point] or ≤ 7 mm [0 point]) + ENS (positive [1 point] or negative [0 point]). The probability of histopathologic metastases was 91.7% when both criteria were met (2 points), 27.1% when one of these criteria was not met (1 point), and 9.0% when neither criterion was met (0 points) (Table 3). The AUC of this new scoring system was 0.846.

**Table 3:** Predictive probability for histopathologic metastases in the scoring system generated in this study

Score	Number of LN metastases (%)	Number of non-LN metastases (%)
Score 0	7 (9.0%)	71 (91.0%)
Score 1	16 (27.1%)	43 (72.9%)
Score 2	33 (91.7%)	3 (8.3%)

### Potential influence of sidedness of colorectal cancer

When the population was subclassified into left and right, in accordance with the location of the primary tumor, the size of 2D LAD for right-sided primary tumors was larger than that for left-sided primary tumors (*p* < 0.005) (median, 8.25 mm; range, 4.55–21.10 mm vs. median, 6.68 mm; range, 4.70–22.05 mm, respectively), while the incidence of histopathologic metastases was similar between right-sided and left-sided primary tumors (37.9% vs. 25.6%, *p* = 0.103). The AUCs for 2D LAD and the scoring system were 0.691 (cut-off value, 7.15 mm) and 0.842, respectively, for right-sided primary tumors, while the AUCs were 0.805 (cut-off value, 7.05 mm) and 0.844, respectively, for left-sided primary tumors.

## Discussion

The current study sought the best CT measure for predicting LN metastases from colorectal cancer. Among the various measures available on MDCT, 2D LAD was the most sensitive measure; moreover, the presence of ENS was associated with LN metastases. Although the baseline sizes of LNs seemed to be larger in right-sided primary tumors, the scoring system using 2D LAD and ENS showed good performance for predicting LN metastases from primary tumors on either side.

Conventionally, a LAD of > 10 mm has been accepted as a potential sign of LN metastases [5, 6]. Reported sensitivities and specificities of LN size and/or clusters of ≥ 3 LNs were 56–84.3% and 58–95%, respectively [5–9]. Dighe et al. demonstrated that studies using ≤ 5-mm-thick CT sections show significantly better results than those using > 5-mm-thick CT sections [13]; moreover, a recent retrospective study reviewed the accuracy of conventional LN size criteria, using recent CT techniques, and reported that the ability of preoperative CT to assess LNs remained unsatisfactory [14].

In the current study, various radiologic measures available in MDCT were compared to establish a valid method for the prediction of LN metastases. Among the tested CT measures, 2D LAD in axial images showed the best performance for the prediction of LN metastases; notably, 3D reconstruction did not show diagnostic superiority to the 2D measures. Interestingly, although the baseline size of LNs seemed to be larger in right-sided primary tumors, the cut-off values for 2D LAD were similar between the right-sided and left-sided primary tumors (7.15 mm vs. 7.05 mm).

The current study also tested the diagnostic accuracy of CT attenuation and size ratio (SAD to LAD). A previous study reported that the cut-off value of 0.8 in size ratio was associated with an increased incidence of LN metastases [15], while another study reported that a measurement of ≥ 100 Hounsfield units was correlated with LN metastases [9]. However, these measures did not show good performance in the present study. However, a new measure, ENS, showed good correlation with LN metastases in this study. ENS indicates a certain type of morphology and is one of the criteria for diagnosing LN metastasis in cervical nodes from head and neck squamous cell carcinoma [12]. A recent retrospective study also reported that the presence of at least one LN with internal heterogeneity and/or irregular outer border is an important predictive factor in colon cancer [16]. Given that 2D LAD and ENS showed good correlation with the incidence of LN metastases, a scoring system was established in this study. This simple score, using a 7-mm cut-off value for 2D LAD, along with the presence or absence of ENS, showed good predictive performance for LN metastases, even in the subgroup analysis stratified by the sidedness of the primary tumor.

The limitations of this study include its retrospective nature and the relatively small patient sample size. However, the current analysis used prospectively corrected CT findings measured by two independent radiologists; moreover, a sufficient number of LNs were studied, with a one-to-one correlation of the LNs detected on CT to those confirmed pathologically. Although validation studies using a larger population are needed, the current results may offer a simple and potentially reliable method to predict LN metastases in colorectal cancer.

## Conclusions

In conclusion, 2D LAD shows the best performance in predicting LN metastases; evaluation for the presence of ENS further improves the diagnostic accuracy for predicting LN metastases prior to surgery. A prospective study to validate the current simple scoring system, using 2D LAD and ENS, is therefore warranted.

## Abbreviations

AUC: Area under the curve
CT: Computed tomography
ENS: Extranodal neoplastic spread
LAD: Long-axis diameter
LN: Lymph node
MDCT: Multidetector-row computed tomography
ROC: Receiver operating characteristic
SAD: Short-axis diameter
